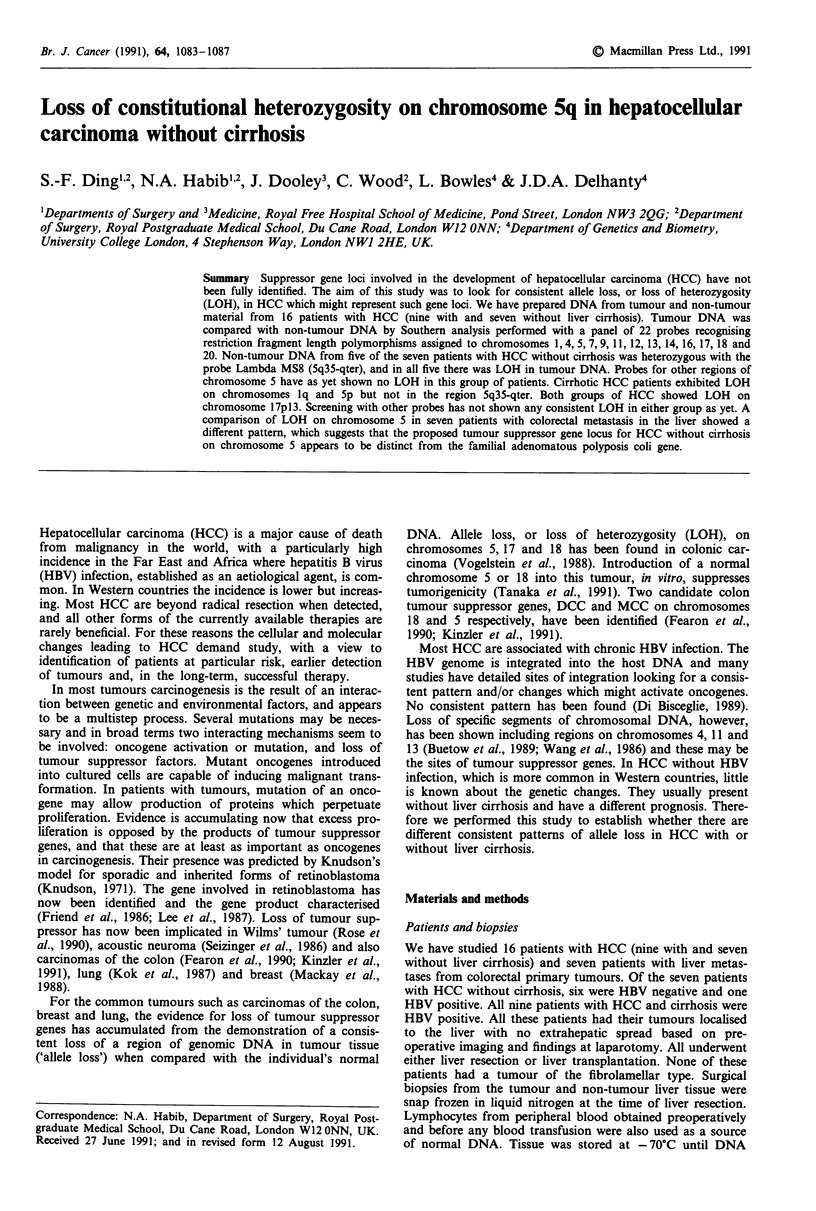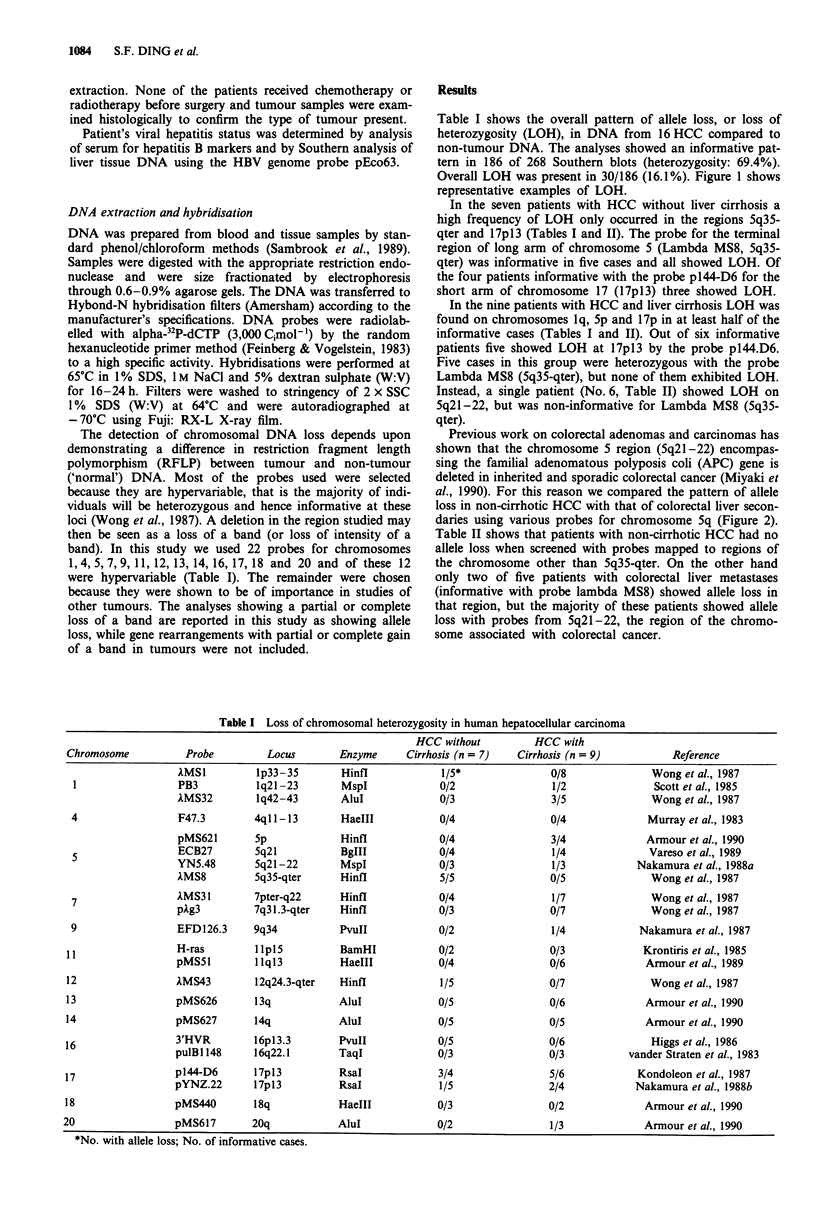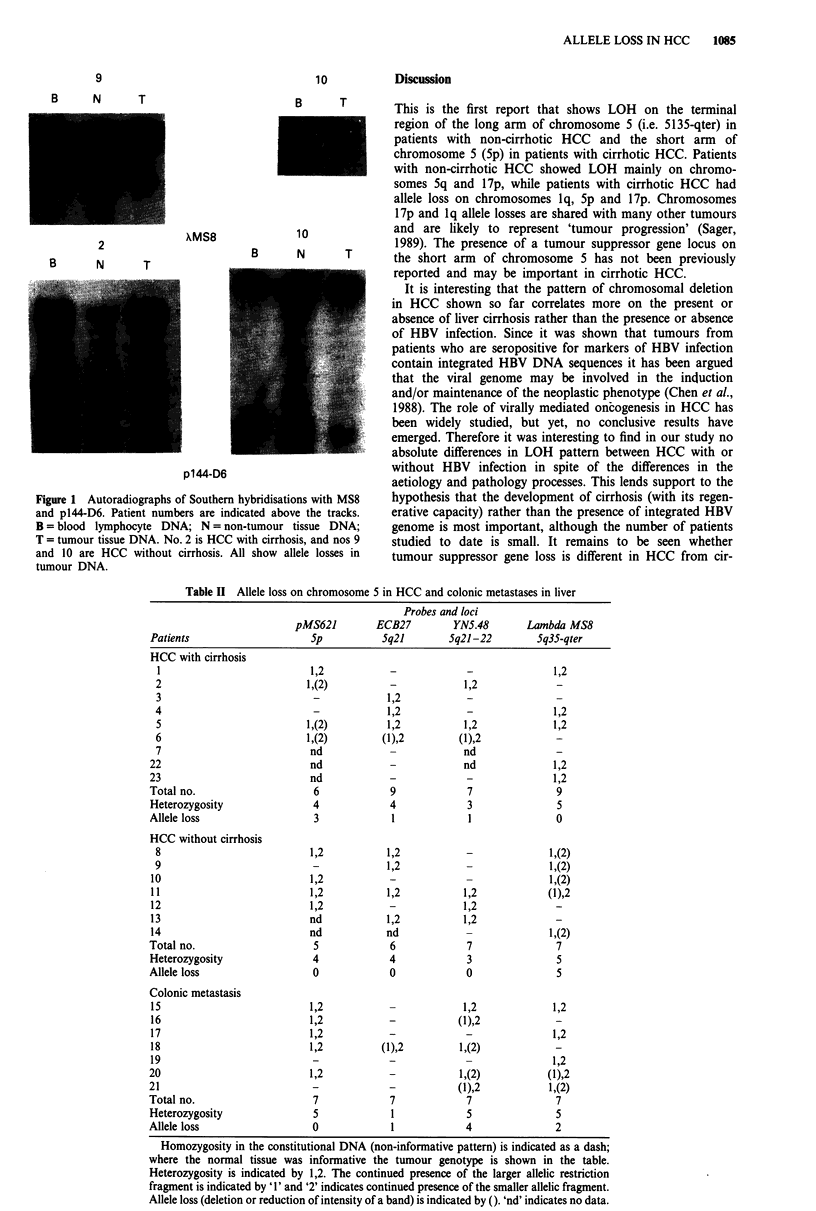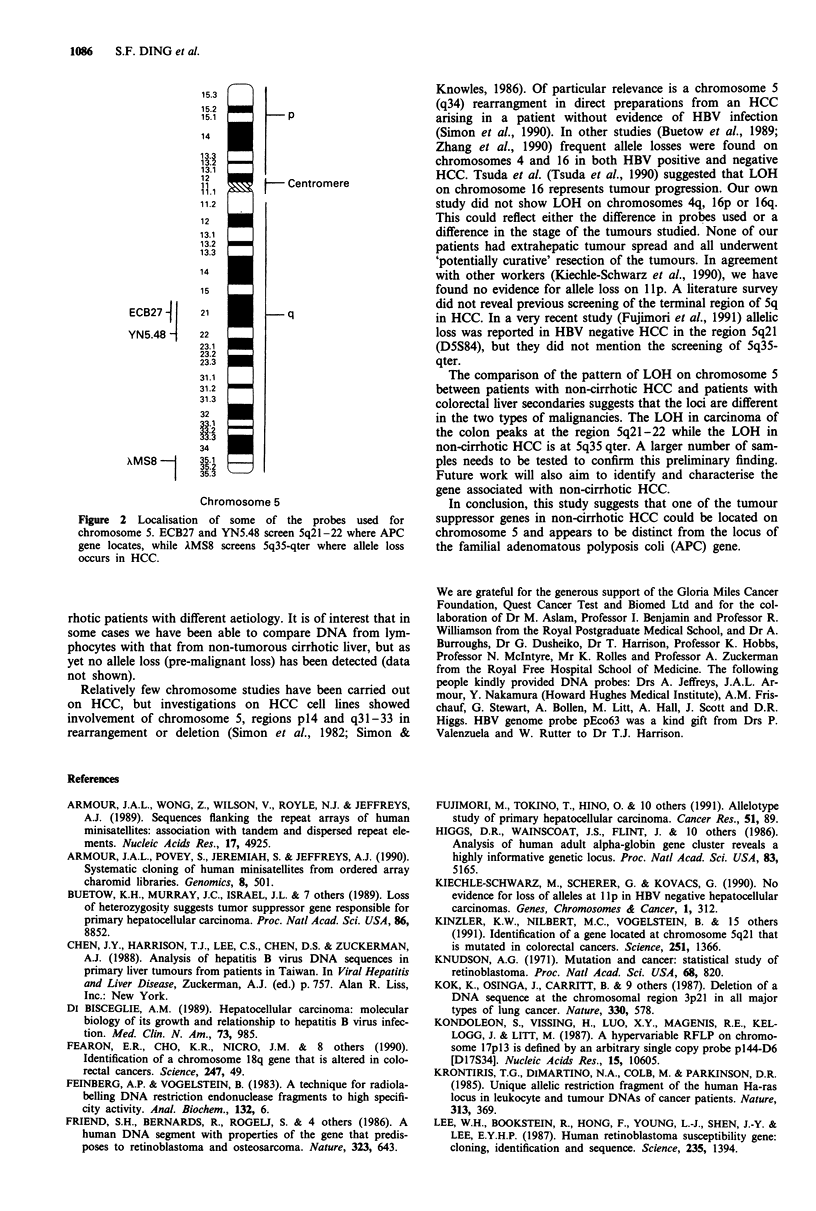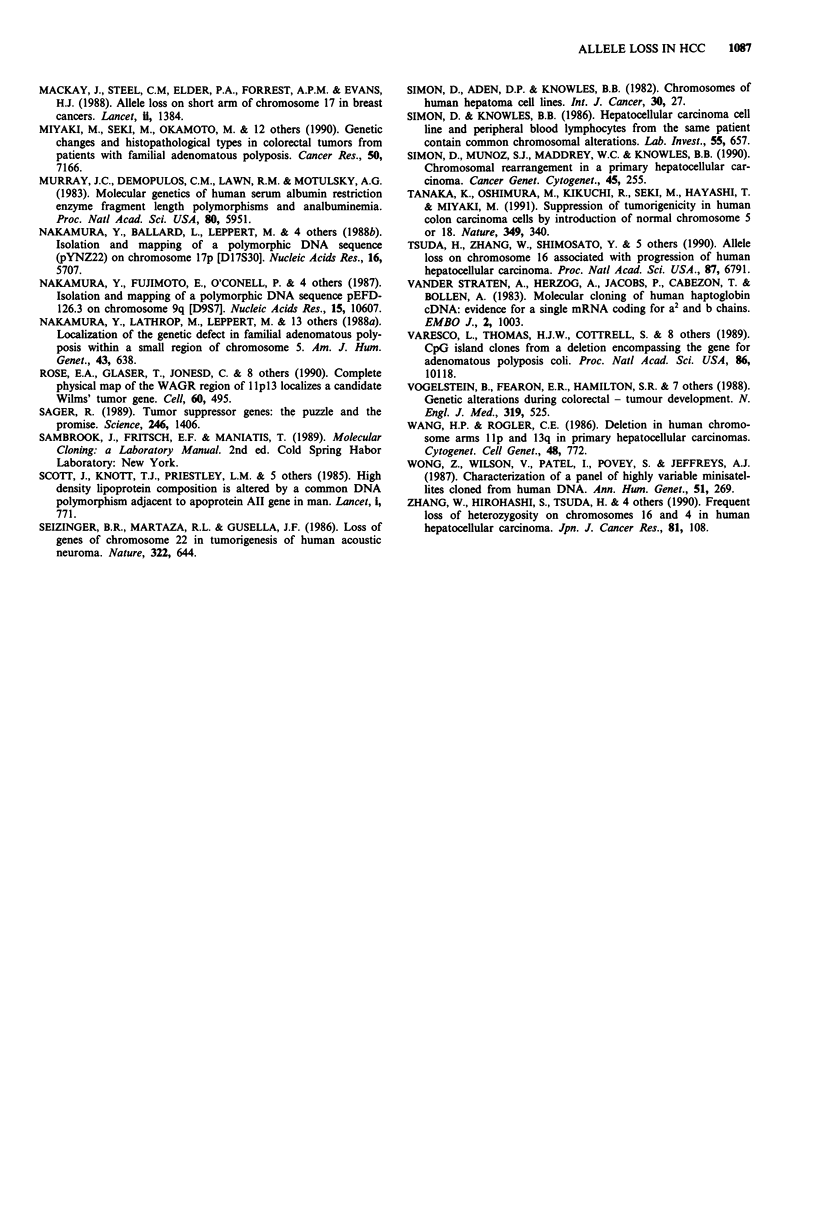# Loss of constitutional heterozygosity on chromosome 5q in hepatocellular carcinoma without cirrhosis.

**DOI:** 10.1038/bjc.1991.468

**Published:** 1991-12

**Authors:** S. F. Ding, N. A. Habib, J. Dooley, C. Wood, L. Bowles, J. D. Delhanty

**Affiliations:** Department of Surgery, Royal Free Hospital School of Medicine, London, UK.

## Abstract

**Images:**


*Br. J. Cancer* (1991), **64**, 1083–1087 © Macmillan Press Ltd., 1991

### Loss of constitutional heterozygosity on chromosome 5q in hepatocellular carcinoma without cirrhosis

S.-F. Ding[1,2], N.A. Habib[1,2], J. Dooley[3], C. Wood[2], L. Bowles[4] & J.D.A. Delhanty[4]

[1]*Departments of Surgery and* [3]*Medicine, Royal Free Hospital School of Medicine, Pond Street, London NW3 2QG;* [2]*Department of Surgery, Royal Postgraduate Medical School, Du Cane Road, London W12 0NN;* [4]*Department of Genetics and Biometry, University College London, 4 Stephenson Way, London NW1 2HE, UK.*

**Summary** Suppressor gene loci involved in the development of hepatocellular carcinoma (HCC) have not been fully identified. The aim of this study was to look for consistent allele loss, or loss of heterozygosity (LOH), in HCC which might represent such gene loci. We have prepared DNA from tumour and non-tumour material from 16 patients with HCC (nine with and seven without liver cirrhosis). Tumour DNA was compared with non-tumour DNA by Southern analysis performed with a panel of 22 probes recognising restriction fragment length polymorphisms assigned to chromosomes 1, 4, 5, 7, 9, 11, 12, 13, 14, 16, 17, 18 and 20. Non-tumour DNA from five of the seven patients with HCC without cirrhosis was heterozygous with the probe Lambda MS8 (5q35-qter), and in all five there was LOH in tumour DNA. Probes for other regions of chromosome 5 have as yet shown no LOH in this group of patients. Cirrhotic HCC patients exhibited LOH on chromosomes 1q and 5p but not in the region 5q35-qter. Both groups of HCC showed LOH on chromosome 17p13. Screening with other probes has not shown any consistent LOH in either group as yet. A comparison of LOH on chromosome 5 in seven patients with colorectal metastasis in the liver showed a different pattern, which suggests that the proposed tumour suppressor gene locus for HCC without cirrhosis on chromosome 5 appears to be distinct from the familial adenomatous polyposis coli gene.

Hepatocellular carcinoma (HCC) is a major cause of death from malignancy in the world, with a particularly high incidence in the Far East and Africa where hepatitis B virus (HBV) infection, established as an aetiological agent, is common. In Western countries the incidence is lower but increasing. Most HCC are beyond radical resection when detected, and all other forms of the currently available therapies are rarely beneficial. For these reasons the cellular and molecular changes leading to HCC demand study, with a view to identification of patients at particular risk, earlier detection of tumours and, in the long-term, successful therapy.

In most tumours carcinogenesis is the result of an interaction between genetic and environmental factors, and appears to be a multistep process. Several mutations may be necessary and in broad terms two interacting mechanisms seem to be involved: oncogene activation or mutation, and loss of tumour suppressor factors. Mutant oncogenes introduced into cultured cells are capable of inducing malignant transformation. In patients with tumours, mutation of an oncogene may allow production of proteins which perpetuate proliferation. Evidence is accumulating now that excess proliferation is opposed by the products of tumour suppressor genes, and that these are at least as important as oncogenes in carcinogenesis. Their presence was predicted by Knudson's model for sporadic and inherited forms of retinoblastoma (Knudson, 1971). The gene involved in retinoblastoma has now been identified and the gene product characterised (Friend *et al.*, 1986; Lee *et al.*, 1987). Loss of tumour suppressor has now been implicated in Wilms' tumour (Rose *et al.*, 1990), acoustic neuroma (Seizinger *et al.*, 1986) and also carcinomas of the colon (Fearon *et al.*, 1990; Kinzler *et al.*, 1991), lung (Kok *et al.*, 1987) and breast (Mackay *et al.*, 1988).

For the common tumours such as carcinomas of the colon, breast and lung, the evidence for loss of tumour suppressor genes has accumulated from the demonstration of a consistent loss of a region of genomic DNA in tumour tissue ('allele loss') when compared with the individual's normal DNA. Allele loss, or loss of heterozygosity (LOH), on chromosomes 5, 17 and 18 has been found in colonic carcinoma (Vogelstein *et al.*, 1988). Introduction of a normal chromosome 5 or 18 into this tumour, *in vitro*, suppresses tumorigenicity (Tanaka *et al.*, 1991). Two candidate colon tumour suppressor genes, DCC and MCC on chromosomes 18 and 5 respectively, have been identified (Fearon *et al.*, 1990; Kinzler *et al.*, 1991).

Most HCC are associated with chronic HBV infection. The HBV genome is integrated into the host DNA and many studies have detailed sites of integration looking for a consistent pattern and/or changes which might activate oncogenes. No consistent pattern has been found (Di Bisceglie, 1989). Loss of specific segments of chromosomal DNA, however, has been shown including regions on chromosomes 4, 11 and 13 (Buetow *et al.*, 1989; Wang *et al.*, 1986) and these may be the sites of tumour suppressor genes. In HCC without HBV infection, which is more common in Western countries, little is known about the genetic changes. They usually present without liver cirrhosis and have a different prognosis. Therefore we performed this study to establish whether there are different consistent patterns of allele loss in HCC with or without liver cirrhosis.

### Materials and methods

### *Patients and biopsies*

We have studied 16 patients with HCC (nine with and seven without liver cirrhosis) and seven patients with liver metastases from colorectal primary tumours. Of the seven patients with HCC without cirrhosis, six were HBV negative and one HBV positive. All nine patients with HCC and cirrhosis were HBV positive. All these patients had their tumours localised to the liver with no extrahepatic spread based on preoperative imaging and findings at laparotomy. All underwent either liver resection or liver transplantation. None of these patients had a tumour of the fibrolamellar type. Surgical biopsies from the tumour and non-tumour liver tissue were snap frozen in liquid nitrogen at the time of liver resection. Lymphocytes from peripheral blood obtained preoperatively and before any blood transfusion were also used as a source of normal DNA. Tissue was stored at $-70°C$ until DNA

Correspondence: N.A. Habib, Department of Surgery, Royal Postgraduate Medical School, Du Cane Road, London W12 0NN, UK.
Received 27 June 1991; and in revised form 12 August 1991.

extraction. None of the patients received chemotherapy or radiotherapy before surgery and tumour samples were examined histologically to confirm the type of tumour present.

Patient's viral hepatitis status was determined by analysis of serum for hepatitis B markers and by Southern analysis of liver tissue DNA using the HBV genome probe pEco63.

*DNA extraction and hybridisation*

DNA was prepared from blood and tissue samples by standard phenol/chloroform methods (Sambrook *et al.*, 1989). Samples were digested with the appropriate restriction endonuclease and were size fractionated by electrophoresis through 0.6–0.9% agarose gels. The DNA was transferred to Hybond-N hybridisation filters (Amersham) according to the manufacturer's specifications. DNA probes were radiolabelled with alpha-$^{32}$P-dCTP (3,000 $C_i mol^{-1}$) by the random hexanucleotide primer method (Feinberg & Vogelstein, 1983) to a high specific activity. Hybridisations were performed at 65°C in 1% SDS, 1 M NaCl and 5% dextran sulphate (W:V) for 16–24 h. Filters were washed to stringency of 2 × SSC 1% SDS (W:V) at 64°C and were autoradiographed at −70°C using Fuji: RX-L X-ray film.

The detection of chromosomal DNA loss depends upon demonstrating a difference in restriction fragment length polymorphism (RFLP) between tumour and non-tumour ('normal') DNA. Most of the probes used were selected because they are hypervariable, that is the majority of individuals will be heterozygous and hence informative at these loci (Wong *et al.*, 1987). A deletion in the region studied may then be seen as a loss of a band (or loss of intensity of a band). In this study we used 22 probes for chromosomes 1, 4, 5, 7, 9, 11, 12, 13, 14, 16, 17, 18 and 20 and of these 12 were hypervariable (Table I). The remainder were chosen because they were shown to be of importance in studies of other tumours. The analyses showing a partial or complete loss of a band are reported in this study as showing allele loss, while gene rearrangements with partial or complete gain of a band in tumours were not included.

### Results

Table I shows the overall pattern of allele loss, or loss of heterozygosity (LOH), in DNA from 16 HCC compared to non-tumour DNA. The analyses showed an informative pattern in 186 of 268 Southern blots (heterozygosity: 69.4%). Overall LOH was present in 30/186 (16.1%). Figure 1 shows representative examples of LOH.

In the seven patients with HCC without liver cirrhosis a high frequency of LOH only occurred in the regions 5q35-qter and 17p13 (Tables I and II). The probe for the terminal region of long arm of chromosome 5 (Lambda MS8, 5q35-qter) was informative in five cases and all showed LOH. Of the four patients informative with the probe p144-D6 for the short arm of chromosome 17 (17p13) three showed LOH.

In the nine patients with HCC and liver cirrhosis LOH was found on chromosomes 1q, 5p and 17p in at least half of the informative cases (Tables I and II). Out of six informative patients five showed LOH at 17p13 by the probe p144.D6. Five cases in this group were heterozygous with the probe Lambda MS8 (5q35-qter), but none of them exhibited LOH. Instead, a single patient (No. 6, Table II) showed LOH on 5q21–22, but was non-informative for Lambda MS8 (5q35-qter).

Previous work on colorectal adenomas and carcinomas has shown that the chromosome 5 region (5q21–22) encompassing the familial adenomatous polyposis coli (APC) gene is deleted in inherited and sporadic colorectal cancer (Miyaki *et al.*, 1990). For this reason we compared the pattern of allele loss in non-cirrhotic HCC with that of colorectal liver secondaries using various probes for chromosome 5q (Figure 2). Table II shows that patients with non-cirrhotic HCC had no allele loss when screened with probes mapped to regions of the chromosome other than 5q35-qter. On the other hand only two of five patients with colorectal liver metastases (informative with probe lambda MS8) showed allele loss in that region, but the majority of these patients showed allele loss with probes from 5q21–22, the region of the chromosome associated with colorectal cancer.

**Table I** Loss of chromosomal heterozygosity in human hepatocellular carcinoma

| *Chromosome* | *Probe* | *Locus* | *Enzyme* | *HCC without Cirrhosis (n = 7)* | *HCC with Cirrhosis (n = 9)* | *Reference* |
|---|---|---|---|---|---|---|
| | λMS1 | 1p33–35 | HinfI | 1/5* | 0/8 | Wong *et al.*, 1987 |
| 1 | PB3 | 1q21–23 | MspI | 0/2 | 1/2 | Scott *et al.*, 1985 |
| | λMS32 | 1q42–43 | AluI | 0/3 | 3/5 | Wong *et al.*, 1987 |
| 4 | F47.3 | 4q11–13 | HaeIII | 0/4 | 0/4 | Murray *et al.*, 1983 |
| | pMS621 | 5p | HinfI | 0/4 | 3/4 | Armour *et al.*, 1990 |
| 5 | ECB27 | 5q21 | BglII | 0/4 | 1/4 | Vareso *et al.*, 1989 |
| | YN5.48 | 5q21–22 | MspI | 0/3 | 1/3 | Nakamura *et al.*, 1988*a* |
| | λMS8 | 5q35-qter | HinfI | 5/5 | 0/5 | Wong *et al.*, 1987 |
| 7 | λMS31 | 7pter-q22 | HinfI | 0/4 | 1/7 | Wong *et al.*, 1987 |
| | pλg3 | 7q31.3-qter | HinfI | 0/3 | 0/7 | Wong *et al.*, 1987 |
| 9 | EFD126.3 | 9q34 | PvuII | 0/2 | 1/4 | Nakamura *et al.*, 1987 |
| 11 | H-ras | 11p15 | BamHI | 0/2 | 0/3 | Krontiris *et al.*, 1985 |
| | pMS51 | 11q13 | HaeIII | 0/4 | 0/6 | Armour *et al.*, 1989 |
| 12 | λMS43 | 12q24.3-qter | HinfI | 1/5 | 0/7 | Wong *et al.*, 1987 |
| 13 | pMS626 | 13q | AluI | 0/5 | 0/6 | Armour *et al.*, 1990 |
| 14 | pMS627 | 14q | AluI | 0/5 | 0/5 | Armour *et al.*, 1990 |
| 16 | 3'HVR | 16p13.3 | PvuII | 0/5 | 0/6 | Higgs *et al.*, 1986 |
| | pulB1148 | 16q22.1 | TaqI | 0/3 | 0/3 | vander Straten *et al.*, 1983 |
| 17 | p144-D6 | 17p13 | RsaI | 3/4 | 5/6 | Kondoleon *et al.*, 1987 |
| | pYNZ.22 | 17p13 | RsaI | 1/5 | 2/4 | Nakamura *et al.*, 1988*b* |
| 18 | pMS440 | 18q | HaeIII | 0/3 | 0/2 | Armour *et al.*, 1990 |
| 20 | pMS617 | 20q | AluI | 0/2 | 1/3 | Armour *et al.*, 1990 |

*No. with allele loss; No. of informative cases.

**Figure 1** Autoradiographs of Southern hybridisations with MS8 and p144-D6. Patient numbers are indicated above the tracks. **B** = blood lymphocyte DNA; **N** = non-tumour tissue DNA; **T** = tumour tissue DNA. No. 2 is HCC with cirrhosis, and nos 9 and 10 are HCC without cirrhosis. All show allele losses in tumour DNA.

### Discussion

This is the first report that shows LOH on the terminal region of the long arm of chromosome 5 (i.e. 5135-qter) in patients with non-cirrhotic HCC and the short arm of chromosome 5 (5p) in patients with cirrhotic HCC. Patients with non-cirrhotic HCC showed LOH mainly on chromosomes 5q and 17p, while patients with cirrhotic HCC had allele loss on chromosomes 1q, 5p and 17p. Chromosomes 17p and 1q allele losses are shared with many other tumours and are likely to represent 'tumour progression' (Sager, 1989). The presence of a tumour suppressor gene locus on the short arm of chromosome 5 has not been previously reported and may be important in cirrhotic HCC.

It is interesting that the pattern of chromosomal deletion in HCC shown so far correlates more on the present or absence of liver cirrhosis rather than the presence or absence of HBV infection. Since it was shown that tumours from patients who are seropositive for markers of HBV infection contain integrated HBV DNA sequences it has been argued that the viral genome may be involved in the induction and/or maintenance of the neoplastic phenotype (Chen *et al.*, 1988). The role of virally mediated oncogenesis in HCC has been widely studied, but yet, no conclusive results have emerged. Therefore it was interesting to find in our study no absolute differences in LOH pattern between HCC with or without HBV infection in spite of the differences in the aetiology and pathology processes. This lends support to the hypothesis that the development of cirrhosis (with its regenerative capacity) rather than the presence of integrated HBV genome is most important, although the number of patients studied to date is small. It remains to be seen whether tumour suppressor gene loss is different in HCC from cir-

**Table II** Allele loss on chromosome 5 in HCC and colonic metastases in liver

| | *Probes and loci* | | | |
|---|---|---|---|---|
| *Patients* | *pMS621 5p* | *ECB27 5q21* | *YN5.48 5q21–22* | *Lambda MS8 5q35-qter* |
| HCC with cirrhosis | | | | |
| 1 | 1,2 | – | – | 1,2 |
| 2 | 1,(2) | – | 1,2 | – |
| 3 | – | 1,2 | – | – |
| 4 | – | 1,2 | – | 1,2 |
| 5 | 1,(2) | 1,2 | 1,2 | 1,2 |
| 6 | 1,(2) | (1),2 | (1),2 | – |
| 7 | nd | – | nd | – |
| 22 | nd | – | nd | 1,2 |
| 23 | nd | – | – | 1,2 |
| Total no. | 6 | 9 | 7 | 9 |
| Heterozygosity | 4 | 4 | 3 | 5 |
| Allele loss | 3 | 1 | 1 | 0 |
| HCC without cirrhosis | | | | |
| 8 | 1,2 | 1,2 | – | 1,(2) |
| 9 | – | 1,2 | – | 1,(2) |
| 10 | 1,2 | – | – | 1,(2) |
| 11 | 1,2 | 1,2 | 1,2 | (1),2 |
| 12 | 1,2 | – | 1,2 | – |
| 13 | nd | 1,2 | 1,2 | – |
| 14 | nd | nd | – | 1,(2) |
| Total no. | 5 | 6 | 7 | 7 |
| Heterozygosity | 4 | 4 | 3 | 5 |
| Allele loss | 0 | 0 | 0 | 5 |
| Colonic metastasis | | | | |
| 15 | 1,2 | – | 1,2 | 1,2 |
| 16 | 1,2 | – | (1),2 | – |
| 17 | 1,2 | – | – | 1,2 |
| 18 | 1,2 | (1),2 | 1,(2) | – |
| 19 | – | – | – | 1,2 |
| 20 | 1,2 | – | 1,(2) | (1),2 |
| 21 | – | – | (1),2 | 1,(2) |
| Total no. | 7 | 7 | 7 | 7 |
| Heterozygosity | 5 | 1 | 5 | 5 |
| Allele loss | 0 | 1 | 4 | 2 |

Homozygosity in the constitutional DNA (non-informative pattern) is indicated as a dash; where the normal tissue was informative the tumour genotype is shown in the table. Heterozygosity is indicated by 1,2. The continued presence of the larger allelic restriction fragment is indicated by '1' and '2' indicates continued presence of the smaller allelic fragment. Allele loss (deletion or reduction of intensity of a band) is indicated by ( ). 'nd' indicates no data.

**Figure 2** Localisation of some of the probes used for chromosome 5. ECB27 and YN5.48 screen 5q21–22 where APC gene locates, while λMS8 screens 5q35-qter where allele loss occurs in HCC.

rhotic patients with different aetiology. It is of interest that in some cases we have been able to compare DNA from lymphocytes with that from non-tumorous cirrhotic liver, but as yet no allele loss (pre-malignant loss) has been detected (data not shown).

Relatively few chromosome studies have been carried out on HCC, but investigations on HCC cell lines showed involvement of chromosome 5, regions p14 and q31–33 in rearrangement or deletion (Simon *et al.*, 1982; Simon & Knowles, 1986). Of particular relevance is a chromosome 5 (q34) rearrangment in direct preparations from an HCC arising in a patient without evidence of HBV infection (Simon *et al.*, 1990). In other studies (Buetow *et al.*, 1989; Zhang *et al.*, 1990) frequent allele losses were found on chromosomes 4 and 16 in both HBV positive and negative HCC. Tsuda *et al.* (Tsuda *et al.*, 1990) suggested that LOH on chromosome 16 represents tumour progression. Our own study did not show LOH on chromosomes 4q, 16p or 16q. This could reflect either the difference in probes used or a difference in the stage of the tumours studied. None of our patients had extrahepatic tumour spread and all underwent 'potentially curative' resection of the tumours. In agreement with other workers (Kiechle-Schwarz *et al.*, 1990), we have found no evidence for allele loss on 11p. A literature survey did not reveal previous screening of the terminal region of 5q in HCC. In a very recent study (Fujimori *et al.*, 1991) allelic loss was reported in HBV negative HCC in the region 5q21 (D5S84), but they did not mention the screening of 5q35-qter.

The comparison of the pattern of LOH on chromosome 5 between patients with non-cirrhotic HCC and patients with colorectal liver secondaries suggests that the loci are different in the two types of malignancies. The LOH in carcinoma of the colon peaks at the region 5q21–22 while the LOH in non-cirrhotic HCC is at 5q35 qter. A larger number of samples needs to be tested to confirm this preliminary finding. Future work will also aim to identify and characterise the gene associated with non-cirrhotic HCC.

In conclusion, this study suggests that one of the tumour suppressor genes in non-cirrhotic HCC could be located on chromosome 5 and appears to be distinct from the locus of the familial adenomatous polyposis coli (APC) gene.

We are grateful for the generous support of the Gloria Miles Cancer Foundation, Quest Cancer Test and Biomed Ltd and for the collaboration of Dr M. Aslam, Professor I. Benjamin and Professor R. Williamson from the Royal Postgraduate Medical School, and Dr A. Burroughs, Dr G. Dusheiko, Dr T. Harrison, Professor K. Hobbs, Professor N. McIntyre, Mr K. Rolles and Professor A. Zuckerman from the Royal Free Hospital School of Medicine. The following people kindly provided DNA probes: Drs A. Jeffreys, J.A.L. Armour, Y. Nakamura (Howard Hughes Medical Institute), A.M. Frischauf, G. Stewart, A. Bollen, M. Litt, A. Hall, J. Scott and D.R. Higgs. HBV genome probe pEco63 was a kind gift from Drs P. Valenzuela and W. Rutter to Dr T.J. Harrison.

### References

ARMOUR, J.A.L., WONG, Z., WILSON, V., ROYLE, N.J. & JEFFREYS, A.J. (1989). Sequences flanking the repeat arrays of human minisatellites: association with tandem and dispersed repeat elements. *Nucleic Acids Res.*, **17**, 4925.

ARMOUR, J.A.L., POVEY, S., JEREMIAH, S. & JEFFREYS, A.J. (1990). Systematic cloning of human minisatellites from ordered array charomid libraries. *Genomics*, **8**, 501.

BUETOW, K.H., MURRAY, J.C., ISRAEL, J.L. & 7 others (1989). Loss of heterozygosity suggests tumor suppressor gene responsible for primary hepatocellular carcinoma. *Proc. Natl Acad. Sci. USA*, **86**, 8852.

CHEN, J.Y., HARRISON, T.J., LEE, C.S., CHEN, D.S. & ZUCKERMAN, A.J. (1988). Analysis of hepatitis B virus DNA sequences in primary liver tumours from patients in Taiwan. In *Viral Hepatitis and Liver Disease*, Zuckerman, A.J. (ed.) p. 757. Alan R. Liss, Inc.: New York.

DI BISCEGLIE, A.M. (1989). Hepatocellular carcinoma: molecular biology of its growth and relationship to hepatitis B virus infection. *Med. Clin. N. Am.*, **73**, 985.

FEARON, E.R., CHO, K.R., NICRO, J.M. & 8 others (1990). Identification of a chromosome 18q gene that is altered in colorectal cancers. *Science*, **247**, 49.

FEINBERG, A.P. & VOGELSTEIN, B. (1983). A technique for radiolabelling DNA restriction endonuclease fragments to high specificity activity. *Anal. Biochem.*, **132**, 6.

FRIEND, S.H., BERNARDS, R., ROGELJ, S. & 4 others (1986). A human DNA segment with properties of the gene that predisposes to retinoblastoma and osteosarcoma. *Nature*, **323**, 643.

FUJIMORI, M., TOKINO, T., HINO, O. & 10 others (1991). Allelotype study of primary hepatocellular carcinoma. *Cancer Res.*, **51**, 89.

HIGGS, D.R., WAINSCOAT, J.S., FLINT, J. & 10 others (1986). Analysis of human adult alpha-globin gene cluster reveals a highly informative genetic locus. *Proc. Natl Acad. Sci. USA*, **83**, 5165.

KIECHLE-SCHWARZ, M., SCHERER, G. & KOVACS, G. (1990). No evidence for loss of alleles at 11p in HBV negative hepatocellular carcinomas. *Genes, Chromosomes & Cancer*, **1**, 312.

KINZLER, K.W., NILBERT, M.C., VOGELSTEIN, B. & 15 others (1991). Identification of a gene located at chromosome 5q21 that is mutated in colorectal cancers. *Science*, **251**, 1366.

KNUDSON, A.G. (1971). Mutation and cancer: statistical study of retinoblastoma. *Proc. Natl Acad. Sci. USA*, **68**, 820.

KOK, K., OSINGA, J., CARRITT, B. & 9 others (1987). Deletion of a DNA sequence at the chromosomal region 3p21 in all major types of lung cancer. *Nature*, **330**, 578.

KONDOLEON, S., VISSING, H., LUO, X.Y., MAGENIS, R.E., KELLOGG, J. & LITT, M. (1987). A hypervariable RFLP on chromosome 17p13 is defined by an arbitrary single copy probe p144-D6 [D17S34]. *Nucleic Acids Res.*, **15**, 10605.

KRONTIRIS, T.G., DIMARTINO, N.A., COLB, M. & PARKINSON, D.R. (1985). Unique allelic restriction fragment of the human Ha-ras locus in leukocyte and tumour DNAs of cancer patients. *Nature*, **313**, 369.

LEE, W.H., BOOKSTEIN, R., HONG, F., YOUNG, L.-J., SHEN, J.-Y. & LEE, E.Y.H.P. (1987). Human retinoblastoma susceptibility gene: cloning, identification and sequence. *Science*, **235**, 1394.

MACKAY, J., STEEL, C.M, ELDER, P.A., FORREST, A.P.M. & EVANS, H.J. (1988). Allele loss on short arm of chromosome 17 in breast cancers. *Lancet*, **ii**, 1384.

MIYAKI, M., SEKI, M., OKAMOTO, M. & 12 others (1990). Genetic changes and histopathological types in colorectal tumors from patients with familial adenomatous polyposis. *Cancer Res.*, **50**, 7166.

MURRAY, J.C., DEMOPULOS, C.M., LAWN, R.M. & MOTULSKY, A.G. (1983). Molecular genetics of human serum albumin restriction enzyme fragment length polymorphisms and analbuminemia. *Proc. Natl Acad. Sci. USA*, **80**, 5951.

NAKAMURA, Y., BALLARD, L., LEPPERT, M. & 4 others (1988*b*). Isolation and mapping of a polymorphic DNA sequence (pYNZ22) on chromosome 17p [D17S30]. *Nucleic Acids Res.*, **16**, 5707.

NAKAMURA, Y., FUJIMOTO, E., O'CONELL, P. & 4 others (1987). Isolation and mapping of a polymorphic DNA sequence pEFD-126.3 on chromosome 9q [D9S7]. *Nucleic Acids Res.*, **15**, 10607.

NAKAMURA, Y., LATHROP, M., LEPPERT, M. & 13 others (1988*a*). Localization of the genetic defect in familial adenomatous polyposis within a small region of chromosome 5. *Am. J. Hum. Genet.*, **43**, 638.

ROSE, E.A., GLASER, T., JONESD, C. & 8 others (1990). Complete physical map of the WAGR region of 11p13 localizes a candidate Wilms' tumor gene. *Cell*, **60**, 495.

SAGER, R. (1989). Tumor suppressor genes: the puzzle and the promise. *Science*, **246**, 1406.

SAMBROOK, J., FRITSCH, E.F. & MANIATIS, T. (1989). *Molecular Cloning: a Laboratory Manual.* 2nd ed. Cold Spring Habor Laboratory: New York.

SCOTT, J., KNOTT, T.J., PRIESTLEY, L.M. & 5 others (1985). High density lipoprotein composition is altered by a common DNA polymorphism adjacent to apoprotein AII gene in man. *Lancet*, **i**, 771.

SEIZINGER, B.R., MARTAZA, R.L. & GUSELLA, J.F. (1986). Loss of genes of chromosome 22 in tumorigenesis of human acoustic neuroma. *Nature*, **322**, 644.

SIMON, D., ADEN, D.P. & KNOWLES, B.B. (1982). Chromosomes of human hepatoma cell lines. *Int. J. Cancer*, **30**, 27.

SIMON, D. & KNOWLES, B.B. (1986). Hepatocellular carcinoma cell line and peripheral blood lymphocytes from the same patient contain common chromosomal alterations. *Lab. Invest.*, **55**, 657.

SIMON, D., MUNOZ, S.J., MADDREY, W.C. & KNOWLES, B.B. (1990). Chromosomal rearrangement in a primary hepatocellular carcinoma. *Cancer Genet. Cytogenet.*, **45**, 255.

TANAKA, K., OSHIMURA, M., KIKUCHI, R., SEKI, M., HAYASHI, T. & MIYAKI, M. (1991). Suppression of tumorigenicity in human colon carcinoma cells by introduction of normal chromosome 5 or 18. *Nature*, **349**, 340.

TSUDA, H., ZHANG, W., SHIMOSATO, Y. & 5 others (1990). Allele loss on chromosome 16 associated with progression of human hepatocellular carcinoma. *Proc. Natl Acad. Sci. USA.*, **87**, 6791.

VANDER STRATEN, A., HERZOG, A., JACOBS, P., CABEZON, T. & BOLLEN, A. (1983). Molecular cloning of human haptoglobin cDNA: evidence for a single mRNA coding for $a^2$ and b chains. *EMBO J.*, **2**, 1003.

VARESCO, L., THOMAS, H.J.W., COTTRELL, S. & 8 others (1989). CpG island clones from a deletion encompassing the gene for adenomatous polyposis coli. *Proc. Natl Acad. Sci. USA*, **86**, 10118.

VOGELSTEIN, B., FEARON, E.R., HAMILTON, S.R. & 7 others (1988). Genetic alterations during colorectal – tumour development. *N. Engl. J. Med.*, **319**, 525.

WANG, H.P. & ROGLER, C.E. (1986). Deletion in human chromosome arms 11p and 13q in primary hepatocellular carcinomas. *Cytogenet. Cell Genet.*, **48**, 772.

WONG, Z., WILSON, V., PATEL, I., POVEY, S. & JEFFREYS, A.J. (1987). Characterization of a panel of highly variable minisatellites cloned from human DNA. *Ann. Hum. Genet.*, **51**, 269.

ZHANG, W., HIROHASHI, S., TSUDA, H. & 4 others (1990). Frequent loss of heterozygosity on chromosomes 16 and 4 in human hepatocellular carcinoma. *Jpn. J. Cancer Res.*, **81**, 108.